# Stability and change in alcohol habits of different socio-demographic subgroups - a cohort study

**DOI:** 10.1186/1471-2458-14-525

**Published:** 2014-05-29

**Authors:** Lovisa Sydén, Peter Wennberg, Yvonne Forsell, Anders Romelsjö

**Affiliations:** 1 Department of Public Health Sciences, Division of Social Medicine, Karolinska Institutet, Widerströmska Huset, floor 8, Tomtebodav. 18 A, SE-171 77 Stockholm, Sweden; 2 Stockholm University, SoRAD, SE-106 91 Stockholm, Sweden; 3 Department of Public Health Sciences, Division of Public Health Epidemiology, Karolinska Institutet, Widerströmska Huset, floor 10, Tomtebodav, 18 A, SE-171 77 Stockholm, Sweden

**Keywords:** Alcohol, Social epidemiology, Socio-economic position, Cohort studies, Public health

## Abstract

**Background:**

Stability in alcohol habits varies over time and in subgroups, but there are few longitudinal studies assessing stability in alcohol habits by socio-demographic subgroups and potential predictors of stability and change. The aim was to study stability and change in alcohol habits by sex, age, and socio-economic position (SEP).

**Methods:**

Data derived from two longitudinal population based studies in Sweden; the PART study comprising 19 457 individuals aged 20-64 years in 1998-2000, and the Stockholm Public Health Cohort (SPHC) with 50 067 individuals aged 18-84 years in 2002. Both cohorts were followed-up twice; PART 2000-2003 and 2010, and SPHC 2007 and 2010. Alcohol habits were measured with the Alcohol Use Disorders Identification Test (AUDIT), and with normal weekly alcohol consumption (NWAC). Stability in alcohol habits was measured with intraclass correlation. Odds ratios were estimated in multinomial logistic regression analysis to predict stability in alcohol habits.

**Results:**

For the two drinking measures there were no consistent patterns of stability in alcohol habits by sex or educational level. The stability was higher for older age groups and self-employed women. To be a man aged 30-39 at baseline predicted both increase and decrease in alcohol habits.

**Conclusions:**

The findings illustrate higher stability in alcohol habits with increasing age and among self-employed women with risky alcohol habits. To be a man and the age 30-39 predicted change in alcohol habits. No conclusive pattern of socio-economic position as predictor of change in alcohol habits was found and other studies of potential predictors seem warranted.

## Background

Stability in alcohol habits seems to vary over time and in different subgroups [1]. A typical drinking pattern in many high income countries includes a debut in teenage with increasing consumption until early adulthood, followed by gradually decreasing consumption [1-3]. Teenagers and young adults also tend to have higher levels of episodic drinking compared to adults with a more continuous consumption [4]. Furthermore, men drink more than women [3] and socio-economic subgroups are complexly associated with alcohol habits and alcohol-related problems and mortality [5-7]. In Sweden alcohol habits are heterogeneous; men drink more than women and the consumption decreases with age, but both of these differences tend to abate [8,9].

Longitudinal studies of alcohol consumption generally find age related changes [1,4,10] often with decreasing consumption or transition to nondrinking with age. Fillmore et al. [11] and Johnstone et al. [2] studied the patterning of change in drinking behaviour across the life course with combined multiple longitudinal data sets. They found an overall modest contribution of gender to variability in pattern of change in drinking, and higher age predicted more stable pattern of drinking. Kerr et al. [12] found lower stability in alcohol consumption for longer follow-ups and in younger samples in three longitudinal population surveys from the United States.

However, the variation in alcohol habits is diverse and although many studies have emphasised declines in alcohol consumption with increasing age more recent cohorts in the United States shows a tendency towards more stable consumption with increasing age [1]. This is supported by a study of men aged 42-60 years in Finland [13], where the weekly alcohol consumption increased in the age group of 42-year olds and remained stable among the older cohorts, and in a longitudinal study of women aged 43 in 1998 in Sweden [14], showing high stability in alcohol consumption with increasing age.

Gender and education have been found to predict changes in alcohol habits, where women are more likely to decrease or quit drinking than men in all ages. Moore et al. [1] found male gender and lower educational level to predict decline in alcohol consumption in the US, which may not be generalizable to e.g. Sweden. They also found that male gender, higher educational level and being employed predicted increased consumption. Molander et al. [4] found gender and education to predict changes in drinking across various drinking measures in Wisconsin. However, research indicate that the declines in drinking with age are more consistent in North America and Europe than elsewhere, and that women’s risky drinking may be associated with lower levels of education in high income countries but with higher levels of education in low-income countries [15,16].

In order to target interventions, it is necessary to have knowledge, not only about changes in alcohol habits in different subgroups, but also which groups that tend to keep their drinking habits stable over time [2,17]. With regard to the theory on collectivity of drinking cultures, we expect our findings to show fairly equal stability of alcohol habits in different socio-demographic subgroups over time [18].

While several studies have described the change of alcohol habits based on the level of alcohol consumption in subgroups, the literature on longitudinal stability in alcohol habits in socio-economic subgroups is sparse [19]. There are few studies assessing how these predictors may influence stability or change in alcohol habits. This paper studies two measures of alcohol habits to give a broader picture of stability in alcohol habits in the studied population. Against this background, the aim was to study stability and change in alcohol habits by sex, age and SEP, measured by educational level and occupational class. Two research questions were formulated: 1) How does stability in alcohol habits differ in subgroups of sex, age and SEP? 2) Do sex, age and SEP predict stability and change in alcohol habits?

## Method

### Study population

In this study, data from two longitudinal population-based postal survey studies, from the County of Stockholm, capital of Sweden during 1998-2010, were studied; the PART study and the Stockholm Public Health Cohort (SPHC). The Ethical Committee at Karolinska Institutet granted ethical approval for the PART study, and the Stockholm regional ethical review board granted ethical approval for the use of SPHC data.

### The PART study

The first data set derives from the longitudinal study in 1998 to 2010 of mental health, work, and relations (Swedish: *Psykisk hälsa, Arbete och RelaTioner*; PART), in Stockholm County, Sweden. The sample frame included 19 457 randomly selected Swedish citizens residing in Stockholm County during the baseline (T_0_) period 1998-2000, aged 20-64 years. At T_0_ a total of 10 341 individuals responded to the questionnaire, a response rate of 53% [20]. At the first follow-up (T_1_) in 2001-2003, the 10 203 still available participants from the baseline were invited to complete a similar questionnaire, and 8 518 individuals (83%) participated [21]. At the second follow-up (T_2_) in 2010, 5 227 (63%) individuals participated.

### The Stockholm Public Health Cohort

The second data set is from a prospective study, the Stockholm Public Health Cohort (SPHC) from the Stockholm County Council public health surveys. In 2002 (T_0_), the sample frame consisted of 50 067 individuals, aged 18-84 years, representing a random sample of the population in the Stockholm County. A total of 31 182 individuals (62%) responded to the questionnaire at baseline (T_0_). In 2007, at the first follow-up (T_1_), 23 794 (76%) subjects participated. At the second follow-up (T_2_), conducted in 2010, 19 327 (80%) subjects participated [22,23].

### Measurements

### The Alcohol Use Disorders Identification Test (AUDIT)

In PART the Alcohol Use Disorders Identification Test (AUDIT) was used to measure alcohol habits with focus on the past 12 months at T_0_, T_1_ and T_2_. AUDIT consists of ten questions, scoring 0-40 points, and was carried out in a self-reported questionnaire. The total AUDIT score reflects the individual’s level of risk related to alcohol (current consumption, dependence symptoms, and current or earlier alcohol-related consequences). A low score indicates low consumption and few alcohol-related consequences, while a high score indicates high consumption and more severe consequences [24]. AUDIT is sensitive to both alcohol problems and hazardous drinking and is therefore suitable for studies in the general population where the prevalence of alcohol problems is lower than in clinical samples. To detect hazardous consumption the cut-off points in AUDIT were set at 8 points for men and 6 points for women [25].

### Normal weekly alcohol consumption (NWAC)

In SPHC, alcohol habits were measured by self-reported typical weekly alcohol consumption during the last year with normal weekly alcohol consumption (NWAC) [26] at T_0_ and T_2_. Alcohol consumption was measured with centilitres at T_0_ and number of glasses at T_2_ per different beverages: strong cider/alcopop, medium-strong beer, strong beer, wine, strong wine and spirits. The NWAC was calculated into grams of 100% alcohol per week. Risk consumption per normal week was defined as drinking 14 or more standard glasses (in Sweden defined as 12 grams 100% alcohol) of alcohol for men and 9 or more for women [27], i.e. 108 grams for women and 168 grams for men.

It is worth to emphasise that AUDIT and NWAC includes different aspects of alcohol habits. Both measures include current alcohol consumption during the last year, but AUDIT additionally covers current and earlier alcohol related problems, with focus on the last year. Because of this, the stability in the two measures is expected to differ and consistent findings for the two measures of stability and predictors of stability and change in alcohol habits seem more profound. The two measures of alcohol habits were therefore studied for the subgroup variables stated below.

### Socio-demographic variables

Age and sex were extracted from registers for both PART and SPHC. In PART the age at baseline, 20-64 years, was categorized into five subgroups. In SPHC the age at baseline, 18-84 years, was divided into seven age groups.

Socio-economic position (SEP), defined as the social and economic factors that influence a group’s position within a society, may affect health behaviour, and in this case alcohol habits [28,29]. Indicators of SEP were educational level, that has been found to affect the stability in alcohol habits in other contexts as mentioned above, and also occupational class, an often used indicator of SEP in Sweden [30] that could explain different alcohol habits in a Swedish population based on occupation. To measure educational level, self-reported data from the questionnaire in PART and register data from Statistics Sweden linked to the samples in SPHC were used. Educational level was defined as the highest level of completed education at the time of the measurement, self-reported or available in register data when sending out the questionnaire. Educational level was divided into three groups: Low = Primary School or less, Intermediate = Secondary School/Gymnasium and High = Post-secondary/University.

Information on occupational class was obtained from the questionnaires, asking for current or previous occupation (not depending on or confuse with current employment) and categorized according to the Swedish socio-economic classification (SEI) [30] into six groups: Unskilled workers, Skilled workers, Lower non-manual employees, Intermediate non-manual employees, Higher non-manual employees and Self-employed. Persons with no identifiable or reached occupation, including students, conscripts, sickness and disability pensioners, was coded as missing, according to SEI.

### Missing data

Non-participation analyses were made after the first two waves (T_0_ and T_1_) of the PART study using data from official registers. Lower participation rates were associated with being male, younger age, low income, low education, non-Nordic origin, being unmarried and having previous psychiatric diagnoses [20,21]. The mean AUDIT score at T_0_ differed significantly between responders (4.56) and non-responders (5.07) at T_1_ (p <0.001). This significant difference was also found between responders and non-responders from T_1_ to T_2_.

In SPHC non-responders were also more likely to be men, of young age, born outside of Sweden, unmarried, have low income and low educational level [22]. The mean NWAC at T_0_ differed significantly between responders (99.46 grams) and non-responders (93.81 grams) at T_2_, (p <0.01). When divided in age groups, the non-responders in the age 18-29 had higher weekly alcohol consumption than the responders, but for older ages the responders drank more than non-responders.

### Statistical analysis

Data from PART and SPHC were analysed and presented separately and then studied in order to find possible consistent subgroup differences in stability of alcohol habits and predictors of change in alcohol habits. The two drinking measures were treated as continuous variables. To estimate stability in alcohol habits, the intraclass correlation coefficients (ICC), with two-way mixed effects model and 95% confidence interval (CI) [31,32] were calculated for the AUDIT score at T_0,_ T_1_ and T_2_ in PART, and NWAC at T_0_ and T_2_ in SPHC. ICC presents the proportion of variance explained by individual scores, where values closer to 1 are more consistent over time. Over shorter periods of time, the ICC is often used and interpreted as an indication of reliability [33]. With two or more measurement points, or when time between observations increases, the ICC can be used to estimate the stability over time [32].

The ICCs and CI were calculated and interpreted within each subgroup variable to assess the degree of stability. The group wise stability for the two cohorts, the mean AUDIT score and mean NWAC at T_0_, and the relative change in mean from T_0_ to T_2_, expressed in percentage, were calculated for the subgroups.

Multinomial logistic regression analyses were carried out to estimate crude and adjusted ORs with 95% CIs in order to examine if sex, age and SEP predicted stable, decreased or increased alcohol habits. Since there was no comparable information on alcohol consumption at first follow-up in SPHC, only the change between baseline (T_0_) and second follow-up (T_2_) was studied and compared in both PART and SPHC. The dependent variable of change in alcohol habits between (T_0_) and (T_2_) was defined as stable (-0.49 to 0.49 SD: reference group), increased (>0.5 SD) or decreased (≤ -0.5 SD) in AUDIT score and NWAC. Independent variables included in the regression analysis were sex, age, educational level and occupational class at baseline. The statistical analyses were carried out using SPSS Statistics version 20.0.

## Results

### Participants

The participants responding at T_0_, T_1_ and T_2_, 5 227 individuals in PART and 19 327 individuals in SPHC, were included in the study. The baseline distribution of SEP and alcohol use by sex in the two samples is described in Table 1. The mean and standard deviation for alcohol habits are presented by sex and age in Table 2. The mean AUDIT scores and mean NWAC did not change substantially over time for the two cohorts. Participants with information on the studied variables in the analyses were available for 4 277 participants (82%) in PART and 16 688 participants (86%) in SPHC.

**Table 1:** Baseline characteristics for the PART study (1998-2000) and the Stockholm Public Health Cohort (2002)

Variables*	**Level**	**Subgroups**	**PART N = 5 227**		**SPHC N = 19 327**	
			**n**	**%**	**n**	**%**
Educational level	Men	Low	260	12.3	1 356	16.4
		Intermediate	886	42.0	3 378	40.7
		High	960	45.5	3 217	38.8
		*Missing*	*2*	*0.1*	*339*	*4.1*
	Women	Low	455	14.6	1 664	15.1
		Intermediate	1 139	36.5	4 440	40.2
		High	1 520	48.7	4 455	40.4
		*Missing*	*5*	*0.2*	*478*	*4.3*
Occupational class	Men	Unskilled workers	200	9.5	1 487	17.9
		Skilled workers	169	8.0	829	10.0
		Lower non-manual employees	193	9.2	763	9.2
		Intermediate non-manual employees	443	21.0	1 907	23.0
		Higher non-manual employees	682	32.4	2 068	24.9
		Self-employed	185	8.8	852	10.3
		*Missing*	*236*	*11.2*	*384*	*4.6*
	Women	Unskilled workers	321	10.3	1 781	16.1
		Skilled workers	127	4.1	1 007	9.1
		Lower non-manual employees	586	18.8	2 127	19.3
		Intermediate non-manual employees	792	25.4	3 045	27.6
		Higher non-manual employees	686	22.0	2 055	18.6
		Self-employed	127	4.1	465	4.2
		*Missing*	*480*	*15.4*	*557*	*5.0*
Abstainers last 12 months		Men	76	3.6	515	6.2
		*Missing*	*4*	*0.2*	*62*	*0.7*
		Women	170	5.5	1 168	10.6
		*Missing*	*4*	*0.1*	*130*	*1.2*
Risk consumers		Men	421	20.0	2 279	27.5
		*Missing*	*23*	*1.1*	*270*	*3.3*
		Women	501	16.1	2 703	24.5
		*Missing*	*59*	*1.9*	*318*	*2.9*

*Variables: Educational level is defined as highest finished education, from the questionnaires in PART and from Statistics Sweden registers, in SPHC. Occupational class is current or previous occupation, self-reported in the questionnaires, and categorized according to Swedish socio-economic classification (29). Abstainers last 12 months are from a dichotomous question in the questionnaires. A risk consumer is defined as men with 8+ points in AUDIT score and women with 6+ points in AUDIT score in PART and defined as drinking 14 or more standard glasses (> = 168 grams 100% alcohol) for men and 9 standard glasses (> = 108 grams 100% alcohol) for women per week in SPHC.

**Table 2:** Alcohol habits presented for the PART study and the Stockholm Public Health Cohort (SPHC)

Alcohol habits*	**Level**	**Subgroups**	**T**_**0**_				**T**_**1**_				**T**_**2**_			
			**n**	**%**	**Mean**	**SD**	**n**	**%**	**Mean**	**SD**	**n**	**%**	**Mean**	**SD**
**PART**	N = 5 227													
**AUDIT score**	Valid cases		5 145	98.4	4.4	3.6	5 173	99.0	4.3	3.5	5 087	97.3	4.2	3.5
	Men	Age 20-29 at T_0_	335	15.9	7.3	4.6	334	15.8	6.8	4.1	335	15.9	5.5	4.0
		Age 30-39 at T_0_	431	20.4	5.7	4.3	428	20.3	5.4	3.8	426	20.2	5.5	4.1
		Age 40-49 at T_0_	445	21.1	5.2	4.0	447	21.2	5.3	3.9	434	20.6	5.2	4.2
		Age 50-59 at T_0_	662	31.4	5.0	4.1	664	31.5	4.8	3.8	651	30.9	4.9	4.0
		Age 60-64 at T_0_	212	10.1	4.2	3.6	214	10.2	4.2	3.6	206	9.8	4.0	3.4
	*Missing*	*Age 20-64 at T*_*0*_	*23*	*1.1*	NA**	*NA*	*21*	*1.0*	*NA*	*NA*	*56*	*2.7*	*NA*	*NA*
	Women	Age 20-29 at T_0_	571	18.3	4.6	3.2	581	18.6	4.3	3.2	571	18.3	3.5	2.7
		Age 30-39 at T_0_	698	22.4	3.5	2.9	701	22.5	3.6	2.9	696	22.3	3.7	3.1
		Age 40-49 at T_0_	697	22.3	3.6	3.0	703	22.5	3.7	3.0	689	22.1	3.7	3.0
		Age 50-59 at T_0_	803	25.7	3.4	2.8	811	26.0	3.4	2.8	799	25.6	3.4	2.8
		Age 60-64 at T_0_	291	9.3	2.8	2.0	290	9.3	2.8	2.1	280	9.0	3.0	2.8
	*Missing*	*Age 20-64 at T*_*0*_	*59*	*1.9*	*NA*	*NA*	*33*	*1.1*	*NA*	*NA*	*84*	*2.7*	*NA*	*NA*
**SPHC**	N = 19 327													
**Normal week consumption**	Valid cases		18 739	97.0	99.5	99.3	*NA*	*NA*	*NA*	*NA*	18 601	96.2	99.8	103.2
	Men	Age 18-29 at T_0_	925	11.2	145.8	138.6					922	11.1	121.3	119.7
		Age 30-39 at T_0_	1 415	17.1	125.6	107.6					1 404	16.9	120.9	110.2
		Age 40-49 at T_0_	1 503	18.1	134.8	124.0					1 492	18.0	141.8	134.1
		Age 50-59 at T_0_	1 948	23.5	142.2	124.8					1 942	23.4	149.7	132.2
		Age 60-64 at T_0_	930	11.2	128.6	116.9					920	11.1	139.7	126.4
		Age 65-74 at T_0_	982	11.8	115.5	105.4					966	11.7	118.4	116.2
		Age 75-84 at T_0_	317	3.8	85.6	87.5					306	3.7	82.9	83.4
	*Missing*	*Age 18-84 at T*_*0*_	*270*	*3.3*	*NA*	*NA*					*338*	*4.1*	*NA*	*NA*
	Women	Age 18-29 at T_0_	1 491	13.5	83.9	87.5					1 476	13.4	62.5	64.4
		Age 30-39 at T_0_	2 252	20.4	71.0	67.1					2 226	20.2	69.8	66.1
		Age 40-49 at T_0_	2 028	18.4	81.4	74.0					2 034	18.4	83.9	77.4
		Age 50-59 at T_0_	2 451	22.2	82.1	69.1					2 436	22.1	88.7	80.6
		Age 60-64 at T_0_	926	8.4	76.2	69.5					920	8.3	84.0	86.5
		Age 65-74 at T_0_	1 105	10.0	62.7	65.0					1 090	9.9	65.3	75.3
		Age 75-84 at T_0_	466	4.2	43.5	52.5					467	4.2	46.0	67.5
	*Missing*	*Age 18-84 at T*_*0*_	*318*	*2.9*	*NA*	*NA*					*388*	*3.5*	*NA*	*NA*

*Alcohol habits in PART are calculated AUDIT score, 0-40 points, with mean and standard deviation. Alcohol habits in SPHC is the normal weekly alcohol consumption (NWAC) in the questionnaires and given in grams of 100% alcohol per week, with mean and standard deviation calculated from consumed volume of strong cider/alcopop, medium-strong beer, strong beer, wine, strong wine and spirits. **NA = Not available in the data.

### Stability in alcohol habits

The stability in AUDIT 1998 to 2010 for the total cohort was ICC = 0.69. For both men and women the stability in AUDIT was higher in older age groups. The subgroups with highest stability were men aged 60-64 at baseline (ICC = 0.81) within the age groups, men that were intermediate non-manual employees (ICC = 0.75) and women that were skilled workers (ICC = 0.78) within the occupational classes. Most of the SEP groups showed moderate stability, but men that were unskilled workers (ICC = 0.55) had low stability. Also, women aged 20-29 years at baseline (ICC = 0.59) showed low stability in alcohol habits. Men with high educational level had higher stability in alcohol habits compared to men with low and intermediate educational levels but for women there was no difference in stability due to educational level. For more details, see Table 3.

**Table 3:** **Stability and change in AUDIT score in the PART study at T**_**0**_**, T**_**1 **_**and T**_**2**_

**Subgroups**		**N**	ICC*	**95% CI**	**Mean audit AT T**_**0**_	**Change (%) mean audit**
						**T**_**0 **_**- T**_**2**_
**Total**		4 277	0.69	0.68-0.70	4.38	-3.28
**Sex**						
	Men	1 782	0.68	0.66-0.70	5.38	-4.36
	Women	2 495	0.66	0.65-0.68	3.66	-2.15
**Age at T**_**0**_						
Men	20-29 years	240	0.62	0.56-0.68	7.25	-22.59
	30-39 years	389	0.62	0.57-0.67	5.58	-3.00
	40-49 years	404	0.66	0.62-0.70	5.08	1.90
	50-59 years	598	0.72	0.68-0.75	4.95	1.18
	60-64 years	151	0.81	0.76-0.85	4.37	-5.15
Women	20-29 years	429	0.59	0.54-0.64	4.65	-23.25
	30-39 years	573	0.65	0.61-0.69	3.61	3.19
	40-49 years	618	0.68	0.65-0.72	3.54	4.85
	50-59 years	700	0.72	0.69-0.75	3.42	2.05
	60-64 years	175	0.70	0.63-0.76	2.79	9.63
**Educational level at T**_**0**_						
Men	Low	215	0.61	0.54-0.68	5.45	-5.97
	Intermediate	728	0.65	0.62-0.69	5.57	-8.14
	High	839	0.73	0.70-0.75	5.19	-0.41
Women	Low	334	0.70	0.65-0.74	3.24	0.92
	Intermediate	882	0.64	0.60-0.67	3.79	-2.54
	High	1 279	0.67	0.65-0.70	3.68	-2.57
**Occupational class at T**_**0**_						
Men	Unskilled workers	187	0.55	0.47-0.63	5.64	-12.61
	Skilled workers	159	0.67	0.60-0.74	6.04	-15.31
	Lower non-manual employees	181	0.62	0.54-0.69	5.62	-6.09
	Intermediate non-manual employees	424	0.75	0.71-0.78	4.97	-0.95
	Higher non-manual employees	656	0.71	0.68-0.74	5.20	-0.62
	Self-employed	175	0.71	0.64-0.77	5.87	-3.41
Women	Unskilled workers	298	0.69	0.64-0.73	3.65	-4.96
	Skilled workers	117	0.78	0.72-0.84	3.49	-0.49
	Lower non-manual employees	564	0.62	0.58-0.66	3.66	-1.94
	Intermediate non-manual employees	746	0.66	0.62-0.69	3.69	-2.54
	Higher non-manual employees	649	0.68	0.64-0.71	3.64	-1.61
	Self-employed	121	0.60	0.51-0.69	3.75	1.76

*Intraclass correlation (ICC) with two-way mixed effects model where people effects are random and measures effects are fixed.

The total stability in NWAC 2002 to 2010 was ICC = 0.62. Men aged 18-39 showed lower stability in alcohol habits compared to older ages. Women aged 18-29 had the lowest stability (ICC = 0.40) and most probable to change (decrease) their alcohol habits, the stability was higher with age and the age 60-64 was highest (ICC = 0.67), compared to the other ages. The stability in NWAC had no straightforward trend by age, although the older age groups had slightly higher stability than the younger. The age group 50-59 years had lower stability than the surrounding age groups for both men and women. There was no difference in alcohol habits for men due to educational level, but women with low educational level had less stable alcohol habits (ICC = 0.51) than higher educated women. For men all occupational classes had low or moderate stability in alcohol habits, except for higher non-manual employees that had higher stability in alcohol habits (ICC = 0.64). For women all occupational classes had low stability, except for self-employed women who had high stability (ICC = 0.68) combined with the highest mean NWAC at baseline. Self-employed men also had the highest mean NWAC at baseline, but were more prone to change their alcohol habits. For more details, see Table 4.

**Table 4:** **Stability and change in normal weekly alcohol consumption (NWAC) in SPHC at T**_**0 **_**and T**_**2**_

**Subgroups**		**n**	ICC*	**95% CI**	**Mean**	**Chance (%)**
					**Nwac**	**Mean nwac**
					**AT T**_**0**_	**T**_**0 **_**- T**_**2**_
**Total**		16 688	0.62	0.61-0.63	101.51	0.74
**Sex**						
	Men	7 128	0.60	0.58-0.61	133.32	1.31
	Women	9 560	0.57	0.56-0.59	77.79	0.00
**Age at T**_**0**_						
Men	18-29 years	728	0.47	0.41-0.52	149.48	-19.60
	30-39 years	1 339	0.53	0.49-0.57	126.39	-4.53
	40-49 years	1 400	0.66	0.63-0.69	136.28	4.96
	50-59 years	1 820	0.61	0.58-0.64	142.71	6.85
	60-64 years	865	0.64	0.60-0.68	128.94	8.36
	65-74 years	892	0.62	0.58-0.66	116.74	3.71
	75-84 years	84	0.69	0.56-0.79	71.80	17.02
Women	18-29 years	1 210	0.40	0.35-0.44	85.09	-26.42
	30-39 years	2 111	0.56	0.53-0.59	71.48	-0.94
	40-49 years	1 931	0.62	0.59-0.65	82.17	3.64
	50-59 years	2 328	0.59	0.56-0.61	83.16	7.54
	60-64 years	868	0.67	0.64-0.71	78.23	7.82
	65-74 years	984	0.62	0.58-0.65	65.56	3.81
	75-84 years	128	0.68	0.57-0.76	40.33	9.05
**Educational level at T**_**0**_						
Men	Low	1 103	0.58	0.54-0.62	124.37	4.55
	Intermediate	3 042	0.59	0.57-0.61	137.09	2.06
	High	2 983	0.62	0.59-0.64	132.77	-0.59
Women	Low	1 337	0.51	0.47-0.55	70.74	-3.67
	Intermediate	4 061	0.58	0.56-0.60	76.42	1.43
	High	4 162	0.59	0.57-0.61	81.40	-0.29
**Occupational class at T**_**0**_						
Men	Unskilled workers	1 303	0.59	0.56-0.63	121.12	3.07
	Skilled workers	720	0.54	0.49-0.59	122.79	4.43
	Lower non-manual employees	685	0.60	0.55-0.65	133.56	-4.08
	Intermediate non-manual employees	1 737	0.59	0.56-0.62	136.61	-2.54
	Higher non-manual employees	1 903	0.64	0.61-0.67	136.04	1.22
	Self-employed	780	0.58	0.53-0.62	149.20	8.87
Women	Unskilled workers	1 526	0.54	0.51-0.58	62.98	-1.35
	Skilled workers	917	0.53	0.49-0.58	71.21	-1.67
	Lower non-manual employees	1 895	0.57	0.54-0.60	77.23	2.00
	Intermediate non-manual employees	2 856	0.56	0.53-0.58	78.43	-0.81
	Higher non-manual employees	1 933	0.58	0.55-0.61	88.51	-0.19
	Self-employed	433	0.68	0.63-0.73	94.36	3.93

*Intraclass correlation (ICC) with two-way mixed effects model where people effects are random and measures effects are fixed.

### Predictors of stability and change in alcohol habits

To be a man and age 20-39 years at baseline, predicted decrease in AUDIT score between T_0_ and T_2_ in PART. To be a man and age 30-39 at baseline, predicted increase in AUDIT score between T_0_ and T_2_, see Table 5. Neither educational level nor occupational class predicted changes in alcohol habits including alcohol-related problems.

**Table 5:** **Multinomial logistic regressions of stability in alcohol habits in the PART study (T**_**0 **_**and T**_**2**_**)**

**Variable**	Stable*		Decrease**				Increase***			
	**n**	**Row**	**n**	**Row**	**OR**_**crude**_^**A**^	**OR**_**adj**_^**B**^	**n**	**Row**	**OR**_**crude**_^**A**^	**OR**_**adj**_^**B**^
		**%**		**%**	**(95% CI)**	**(95% CI)**		**%**	**(95% CI)**	**(95% CI)**
**Total n = 4 277**	3 336	78.0	515	12.0	-	-	426	10.0	**-**	**-**
**Sex**										
Men	1 293	72.6	266	14.9	1.69	1.88	223	12.5	1.74	1.83
					(1.40-2.03)	(1.54-2.29)			(1.42-2.13)	(1.49-2.26)
Women	2 043	81.9	249	10.0	1	1	203	8.1	1	1
**Age at T**_**0**_										
20-29 years	420	62.8	193	28.8	5.04	5.63	56	8.4	1.35	1.48
					(3.23-7.85)	(3.56-8.92)			(0.83-2.20)	(0.90-2.43)
30-39 years	739	76.8	104	10.8	1.54	1.66	119	12.4	1.63	1.75
					(0.98-2.44)	(1.04-2.64)			(1.05-2.54)	(1.12-2.74)
40-49 years	824	80.6	87	8.5	1.16	1.24	111	10.9	1.37	1.46
					(0.73-1.84)	(0.78-1.99)			(0.88-2.13)	(0.93-2.28)
50-59 years	1 079	83.1	106	8.2	1.08	1.09	113	8.7	1.06	1.07
					(0.68-1.70)	(0.69-1.72)			(0.68-1.65)	(0.69-1.67)
60-64 years	274	84.0	25	7.7	1	1	27	8.3	1	1
**Educational level at T**_**0**_										
Low	435	79.2	56	10.2	0.94	1.21	58	10.6	1.08	1.09
					(0.69-1.28)	(0.85-1.72)			(0.79-1.47)	(0.77-1.55)
Intermediate	1 221	75.8	229	14.2	1.37	1.07	160	9.9	1.06	0.91
					(1.13-1.67)	(0.85-1.35)			(0.85-1.32)	(0.71-1.16)
High	1 680	79.3	230	10.9	1	1	208	9.8	1	1
**Occupational class at T**_**0**_										
Unskilled workers	356	73.4	72	14.8	1.49	1.06	57	11.8	1.31	1.34
					(1.09-2.03)	(0.75-1.51)			(0.94-1.83)	(0.93-1.95)
Skilled workers	199	72.1	47	17.0	1.74	1.28	30	10.9	1.23	1.19
					(1.21-2.50)	(0.85-1.91)			(0.80-1.88)	(0.76-1.87)
Lower non-manual employees	583	78.3	90	12.1	1.14	1.07	72	9.7	1.01	1.21
					(0.86-1.51)	(0.77-1.48)			(0.74-1.37)	(0.86-1.70)
Intermediate non-manual employees	933	79.7	128	10.9	1.01	0.95	109	9.3	0.95	1.03
					(0.78-1.30)	(0.73-1.24)			(0.73-1.25)	(0.78-1.36)
Higher non-manual employees	1 037	79.5	141	10.8	1	1	127	9.7	1	1
Self-employed	228	77.0	37	12.5	1.19	1.20	31	10.5	1.11	1.07
					(0.81-1.76)	(0.79-1.80)			(0.73-1.69)	(0.70-1.65)

*Stable alcohol habits, reference group, with -0.49 to 0.49 SD = - 2 to 2 AUDIT scores from T_0_ to T_2_.

**Decreased alcohol habits with (< -0.5 SD) = < - 3 AUDIT scores or lower from T_0_ to T_2_.

***Increased alcohol habits with > 0.5 SD = > 3 AUDIT scores or more from T_0_ to T_2_.

^A^:Crude OR for each separate variable.

^B^:OR for each variable, adjusted for all variables in the model.

Furthermore, to be a man, ages 18-59 and 65-74 years at baseline, low educational level, and self-employment predicted decrease in NWAC between T_0_ and T_2_ in SPHC. Unskilled workers were less likely to decrease than to be stable. To be a man, aged 30-39, and 50-59 years, having low and intermediate education level and being self-employed, predicted an increase in NWAC, see Table 6.

**Table 6:** **Multinomial logistic regressions of stability in normal weekly alcohol consumption in SPHC (T**_**0 **_**and T**_**2**_**)**

**Variable**	Stable*		Decrease**				Increase***			
	**n**	**Row**	**n**	**Row**	**OR**_**crude**_^**A**^	**OR**_**adj**_^**B**^	**n**	**Row**	**OR**_**crude**_^**A**^	**OR**_**adj**_^**B**^
		**%**		**%**	**(95% CI)**	**(95% CI)**		**%**	**(95% CI)**	**(95% CI)**
**Total n = 16 688**	10 451	62.6	3 052	18.3			3 185	19.1	-	
**Sex**										
Men	3 813	53.5	1 603	22.5	1.93	2.02	1 712	24.0	2.02	2.00
					(1.78-2.09)	(1.85-2.19)			(1.87-2.19)	(1.84-2.17)
Women	6 638	69.4	1 449	15.2	1	1	1 473	15.4	1	1
**Age at T**_**0**_										
18-29 years	1 004	51.8	623	32.1	4.37	4.79	311	16.0	1.37	1.47
					(2.77-6.91)	(3.02-7.60)			(0.93-2.02)	(0.99-2.18)
30-39 years	2 109	61.1	681	19.7	2.28	2.42	660	19.1	1.39	1.48
					(1.44-3.59)	(1.53-3.83)			(0.95-2.02)	(1.01-2.16)
40-49 years	2 174	65.3	534	16.0	1.73	1.76	623	18.7	1.27	1.29
					(1.10-2.73)	(1.11-2.80)			(0.87-1.85)	(0.88-1.90)
50-59 years	2 627	63.3	635	15.3	1.70	1.69	886	21.4	1.49	1.48
					(1.08-2.68)	(1.07-2.67)			(1.03-2.17)	(1.02-2.17)
60-64 years	1 156	66.7	242	14.0	1.48	1.37	335	19.3	1.28	1.20
					(0.92-2.35)	(0.86-2.20)			(0.87-1.89)	(0.81-1.77)
65-74 years	1 226	65.4	315	16.8	1.81	1.71	335	17.9	1.21	1.15
					(1.14-2.88)	(1.08-2.73)			(0.82-1.78)	(0.78-1.70)
75-84 years	155	73.1	22	10.4	1	1	35	16.5	1	1
**Educational level at T**_**0**_										
Low	1 464	60.0	484	19.8	1.17	1.31	492	20.2	1.22	1.26
					(1.04-1.32)	(1.13-1.50)			(1.08-1.37)	(1.10-1.45)
Intermediate	4 402	62.0	1 272	17.9	1.02	1.06	1 429	20.1	1.18	1.18
					(0.94-1.12)	(0.96-1.17)			(1.08-1.28)	(1.07-1.30)
High	4 585	64.2	1 296	18.1	1	1	1 264	17.7	1	1
**Occupational class at T**_**0**_										
Unskilled workers	1 772	62.6	499	17.6	0.97	0.80	558	19.7	1.04	0.95
					(0.85-1.10)	(0.69-0.93)			(0.92-1.18)	(0.82-1.09)
Skilled workers	1 008	61.6	305	18.6	1.04	0.95	324	19.8	1.06	0.99
					(0.89-1.21)	(0.80-1.13)			(0.92-1.24)	(0.84-1.16)
Lower non-manual	1 671	64.8	444	17.2	0.91	0.97	465	18.0	0.92	1.00
employees					(0.80-1.04)	(0.84-1.13)			(0.81-1.05)	(0.86-1.15)
Intermediate non-manual employees	2 945	64.1	845	18.4	0.98	1.03	803	17.5	0.90	0.95
					(0.88-1.10)	(0.92-1.16)			(0.81-1.01)	(0.85-1.07)
Higher non-manual	648	53.4	257	21.2	1	1	308	25.4	1	1
employees										
Self-employed	2 407	62.7	702	18.3	1.36	1.27	727	19.0	1.57	1.34
					(1.15-1.61)	(1.07-1.51)			(1.34-1.85)	(1.14-1.58)

*Stable normal weekly alcohol consumption, reference group, with -0.49 to 0.49 SD = - 43.99 to 43.99 grams of 100% alcohol per week from T_0_ to T_2_.

**Decreased normal weekly alcohol consumption with 0.5 SD = < - 44 grams of 100% alcohol per week or more from T_0_ to T_2_.

***Increased normal weekly alcohol consumption with > 0.5 SD = > 44 grams of 100% alcohol per week or more from T_0_ to T_2_.

^A^: Crude OR for each separate variable.

^B^: OR for each variable, adjusted for all variables in the model.

## Discussion

The present study used data from two cohorts from the same geographical area and approximately the same time period, and two different drinking measures to study stability and change in alcohol habits. Alcohol habits, including alcohol consumption and alcohol-related problems, were more stable in general over time compared to the measure of alcohol consumption only. From the somewhat scattered results, we found four patterns of stability in alcohol habits consistent for both drinking measures;

• There were no major differences in stability between men and women

• The stability tended to be higher in older age groups

• No conclusive pattern of stability was found with regard to educational level or occupational class, except for tendencies of high stability in alcohol habits and risky alcohol habits among self-employed women

• To be a man, and the age 30-39 predicted changes, both increase and decrease, in alcohol habits

The findings show an overall modest contribution of sex to variability in pattern of change in drinking, and increasing age predicted more stable pattern of drinking, in line with earlier findings [2,11]. However, Molander et al. [4] found education to predict drinking changes across different drinking measures, which was not found consistently in our study. Socio-economic position did not predict change in alcohol habits for the measurement of alcohol consumption and alcohol-related problems but low educational level and self-employment predicted change in the measurement of alcohol consumption. Thus, studies of other potential predictors of change in alcohol habits including alcohol-related problems seem warranted.

In Sweden, where the welfare system is considered to be strong and gender equity high, the association between heavy drinking and social stratification has been found to be less pronounced in earlier studies [34]. In a study by Grittner et al. [35] the data on Sweden did not find any significant differences for men and women in educational level and the risk of alcohol-related problems. Our data show that the gender gap in alcohol consumption and alcohol-related problems is narrowing, with a slightly increase for women and decrease for men, which is supported in earlier cross-sectional studies both in Sweden and other countries [9,36]. The effects of sex, age, and socio-economic position are not simple and linear, stability and change in both alcohol consumption and related problems and dependence varies with more complex combinations of these variables.

While the theory on the collectivity of drinking cultures [18] would lead us to expect small differences in stability between subgroups, we found it possible to identify some subgroups that are more stable or prone to change their alcohol habits. Relative changes in the mean can explain the subgroup stability partly, see Tables 3 and 4. Self-employed women had the highest mean measure at baseline within occupational class and were the only occupational class showing increase over time. This was seen for both measures, although the ICC was lower and with wider CI in the PART study, which could be due to the low sample. However, this indicates that this group could be at risk of later alcohol-related problems and maybe more vulnerable to this, having less social security as self-employed. Another interesting finding is that the alcohol habits gets more stable with age at a higher level for both men and women. Due to the findings, different targeted interventions could be formed for the groups mentioned above with stable risky alcohol habits or those with risky alcohol habits that are prone to change in order to prevent alcohol-related consequences in the future. Interesting to notice is that the unskilled and skilled (only men) workers, with low stability in both samples, decreased the mean AUDIT score quite substantially between T_0_ and T_2_, which could be due to attained socio-economic position at the follow-up.

The main strengths of this study are the large population-based samples, deriving from the same demographic area and covering the same time period, and the longitudinal design. Furthermore, alcohol habits are measured with two different drinking measures, giving a broader picture of stability in alcohol habits in the studied population. While several studies have examined changes in alcohol consumption for different subgroups by level of consumption [1,12,13,37], or used the intraclass correlation to study reliability of self-reported age of onset of alcohol use [33,38], ICC is relatively seldom employed to study stability and change in alcohol habits. This study uses ICC to estimate stability in alcohol habits, since ICC is sensitive to both shifts in mean and subjects rank order over time.

Some limitations in this study should be noted, first, the non-participation at baseline. In line with other population studies e.g. [9], both PART and SPHC had high non-participation rates at baseline. Based on non-participation analysis from the two samples, there were relatively small differences between participants and non-participants. Although the non-participants at follow-up had slightly lower consumption in total and more hazardous habits than the participants at baseline, we cannot draw conclusions on their stability in alcohol habits. Second, the number of occasions and the time period differed somewhat for the two cohorts, although the studied subgroup variables were the same in the two cohorts. This may affect possible consistency between the results. Due to these differences and that alcohol habits were measured with two different drinking measures, we compared patterns, rather than levels, in ICC between the two samples. Variation in estimates of stability and change between the two cohorts might be associated with the interval between measurements, the percentage of subjects retained across measurements and the frame and characteristics of the alcohol measures. The analysis was also performed when stratifying by risk consumers, consumers and abstainers at baseline, but the same pattern or no pattern due to low sample were found, strengthening our conclusions. There might also be an overlap in participation in both cohorts since they were made separately, but if so probably a very small overlap. No information is available regarding this.

Third, as this study focused on long-term stability it did not embrace oscillations between the surveys. It is likely that the consistency of alcohol habits at two or three points of time might not fully reflect individual stability. Moreover, other unmeasured factors associated with stability in alcohol habits may better predict and explain stability and change in alcohol for subgroups.

The results add to the knowledge of long-term stability in alcohol habits for different socio-demographic subgroups and how the studied factors predict stability and change in alcohol habits. Based on the findings, we suggest targeted public health efforts to prevent future alcohol-related consequences in foremost self-employed women, men born in 1960-1970 and elderly men and women.

## Conclusions

The findings illustrate higher stability of alcohol habits with increasing age and among self-employed women with risky alcohol habits. To be a man and the age 30-39 predicted change, both decrease and increase, in alcohol habits. No conclusive pattern of socio-economic position as predictors of change in alcohol habits was found and other potential predictors of change in alcohol habits seem warranted.

## Competing interests

The authors declare that they have no competing interest.

## Authors’ contributions

LS designed, wrote the manuscript and performed the statistical analyses. PW participated in the design, drafted and iterated the manuscript. AR participated in the design of and iterated the manuscript. YF participated in the design and iterated the manuscript. All authors read and approved the final manuscript.

## Pre-publication history

The pre-publication history for this paper can be accessed here:

http://www.biomedcentral.com/1471-2458/14/525/prepub
